# Optimizing Patient Preparation and Surgical Experience Using eHealth Technology

**DOI:** 10.2196/medinform.4286

**Published:** 2015-09-01

**Authors:** Amy Waller, Kristy Forshaw, Mariko Carey, Sancha Robinson, Ross Kerridge, Anthony Proietto, Rob Sanson-Fisher

**Affiliations:** ^1^ University of Newcastle & Hunter Medical Research Institute Health Behaviour Research Group Callaghan Australia; ^2^ John Hunter Hospital Perioperative Service Newcastle Australia; ^3^ University of Newcastle School of Medicine and Public Health Callaghan Australia; ^4^ Hunter New England Local Health District Cancer Services and Cancer Network Newcastle Australia

**Keywords:** eHealth, perioperative, postoperative, preoperative, surgery

## Abstract

With population growth and aging, it is expected that the demand for surgical services will increase. However, increased complexity of procedures, time pressures on staff, and the demand for a patient-centered approach continue to challenge a system characterized by finite health care resources. Suboptimal care is reported in each phase of surgical care, from the time of consent to discharge and long-term follow-up. Novel strategies are thus needed to address these challenges to produce effective and sustainable improvements in surgical care across the care pathway. The eHealth programs represent a potential strategy for improving the quality of care delivered across various phases of care, thereby improving patient outcomes. This discussion paper describes (1) the key functions of eHealth programs including information gathering, transfer, and exchange; (2) examples of eHealth programs in overcoming challenges to optimal surgical care across the care pathway; and (3) the potential challenges and future directions for implementing eHealth programs in this setting. The eHealth programs are a promising alternative for collecting patient-reported outcome data, providing access to credible health information and strategies to enable patients to take an active role in their own health care, and promote efficient communication between patients and health care providers. However, additional rigorous intervention studies examining the needs of potential role of eHealth programs in augmenting patients’ preparation and recovery from surgery, and subsequent impact on patient outcomes and processes of care are needed to advance the field. Furthermore, evidence for the benefits of eHealth programs in supporting carers and strategies to maximize engagement from end users are needed.

## Global Burden of Surgical Conditions

Approximately 234 million surgical operations take place each year globally. Depending on the procedure, there may be substantial direct costs for consumers, including specialist consultations and hospitalization, postoperative care, and medications, as well as indirect costs, including travel and lost productivity [1]. Personal costs include pain, suffering, and premature mortality. Hospital costs can vary according to the length of stay, surgical procedure performed, and the care needs of the patient [2]. Patients undergoing surgery are increasingly older, often have complex comorbidities, and require more efficient surgical care [3]. It is expected that with population growth and aging, the demand for surgical services will escalate [4-6]. The health system faces considerable pressure to increase the level and quality of surgical care within finite health care resources.

## Demands for High Quality, Patient-Centered Care Across the Surgical Pathway Are Not Being Met

The surgical care pathway is characterized by multiple phases of care, from the decision to have surgery to discharge from hospital and follow-up care. Providing optimal care across the different phases of the surgical pathway has become increasingly challenging, due to the complexity of procedures, increasing time pressures on staff, and the demand for a patient-centered approach [7]. Breakdowns in one phase can affect other phases, which in turn can cause delays, cancellations, and complications. For example, minimum standards for informed consent and decision making are not always achieved [8]. This eventually results in unnecessary or unwanted procedures or preventable harm [9]. Patients report inadequate preparation, resulting in surgical cancellations and delays, undiagnosed medical problems, and anxiety, as well as increased length of hospital stay, analgesic requirements, and cost of surgical care [3,10]. Discharge planning may be compromised by a lack of guidelines and systems in hospitals, poor information recall, or limited involvement of patients in the discharge process, as well as a shortage of caregiver and community resources to support recovery. Patients do not always receive detailed instructions at the time of discharge, and this increases the risk of an unnecessarily prolonged recovery, thereby reducing quality of life and increasing costs [11]. Novel strategies are thus needed to address these challenges to produce effective and sustainable improvements in surgical care across the care pathway.

## Using eHealth to Address Current Challenges Across the Surgical Pathway

### Overview

The World Health Organization defines eHealth as “the transfer of health-related resources and health care by electronic means, including information, support resources, assessments, interventions, and health care records” [12]. Endorsed as part of a strategic plan to improve quality of health care, one of the key recommendations made by the Institute of Medicine was the use of eHealth programs [13]. The eHealth programs have the potential to support care delivery models, engage providers and patients, and deliver self-assessment and self-management tools [14]. The key functions of eHealth programs can be categorized as information gathering, transfer, and exchange. The aim of this discussion paper is to describe these key functions, and outline how such features can be applied to presurgical and postsurgical care. Advantages and challenges posed by the use of eHealth as well as key gaps in the evidence base are discussed.

### Information Gathering

Variation in the type and quality of information obtained by clinicians during clinical interviews occurs as a consequence of time and resource constraints, as well as individual clinicians’ bias [15]. Utilizing self-report assessments of eHealth programs via tablets can improve data integrity by standardizing information collected by clinicians. To reduce complexity and data-collection time, algorithms can be built-in to the software so that items can be auto-populated or skipped based on responses. Programs can be developed so that patients can access and complete assessments outside the clinic environment before surgical consultations.

### Information Transfer

The eHealth programs can connect patients with credible, standard information and support regardless of geographic location, the clinician providing care, or the resources of the institution. A credible single source of information is critical given the quantity and variable quality of information available on the Internet [16]. When evidence-based practice recommendations change, information can be updated easily and quickly. Patients can control the number of times they access eHealth programs and the level of information they search and obtain. Providing information tailored to an individual’s knowledge and preferences reduces anxiety, improves information comprehension, and recall [17].

### Information Exchange

Health information exchanges (ie, electronic health records) are available as a platform for key information to be made available to authorized health care providers across care settings to promote continuity. This is especially relevant for older patients and those with multiple comorbidities, given the range of health care providers they may encounter. For example, health information exchanges have the potential to support the electronic sharing of clinical data across organizations, offering timely and complete medical records at the point of care. Immediate access to medical records or investigation results can increase satisfaction and treatment compliance [18] and reduce medical errors and complaints against services [19].

## Potential of eHealth to Improve Care and Outcomes Across the Surgical Pathway

The phases of surgical care are conceptualized as follows: the “preoperative phase,” which refers to care delivered prior to surgery; the “intraoperative phase” when surgery is performed; and the “postoperative phase,” which is the period from surgery completion/patient recovery to discharge from hospital. Within each phase are critical steps that patients encounter as they progress through the pathway. We have used these steps as a framework to illustrate examples where eHealth programs could improve outcomes in the preoperative and postoperative phases of care.

## Preoperative Care

### Step 1: Enhancing Decision-Making Process and Streamlining Informed Consent

Ideally, patients should have a complete understanding of the risks, benefits, and potential outcomes of the procedure before consent. eHealth programs can augment standard face-to-face informed consent processes by conveying supplementary information, meeting patients’ preferences, and exploring understanding of information once it has been delivered [20]. Evidence-based features, such as decision aids, can be incorporated and accessed by the patient before the consultation to help focus discussions [21]. Nonbiased presentation of the risks and benefits of relevant options, a table of pros and cons for easy comparison, value-clarification exercises, and targeted assessments can help clarify patient understanding, identify gaps in knowledge, and reduce decisional regret [22-24]. Programs can also act as a point of reference for patients to access after the consent consultation to consolidate and re-explore information.

### Step 2: Collecting Medical History Data, Delivering Information, and Optimizing Preoperative Preparation

Traditionally, there has been only a short timeframe for providing perioperative care [25]. More recently, models of care have been employed in which patient assessment, preparation, and discharge planning begin at the time of booking itself [25]. The eHealth programs enable patients to complete their medical history online at home, or in the waiting room before their surgical consultation using a tablet. This information can then be transferred to the provider in real time so that it is readily accessible and clarified by staff at the preoperative consultation. The eHealth programs can also alleviate some of the burden on providers by delivering written and audiovisual information about the potential risks of anesthesia, the procedure, and preparation requirements [26,27]. Preoperative education programs have reduced length of stay, postoperative medication usage, complications, and anxiety [28]. Providing both procedural and sensory information offers additional benefits [29-31].

### Step 3: Streamlining Admission Procedures

Information should be provided to the patient regarding where they need to go in the hospital, dietary and other preparation requirements, and the processes involved from the point of arrival at the hospital to recovering back in the ward after the procedure. Short message services or email can be used to prompt patients about what to bring with them, including consent forms, test and imaging results, medication lists, and Medicare and health fund details. Electronic reminders can also be used to prompt providers to collect specific information from the patient and/or perform a specific clinical action during admission. Electronic reminders can reduce cancellation rates and increase compliance with instructions.

## Postoperative Care

### Step 4: Delivering Individually Tailored Postoperative Care Plans

Nowadays, postoperative hospital stays are becoming increasingly shorter as a consequence of novel interventions, such as minimally invasive techniques and fast-track programs. Although this can increase patient satisfaction and reduce health care utilization and costs, a major disadvantage is that there is less opportunity for patient education [32]. Using tablets, patients can complete symptom assessments electronically, and during recovery the results can be transmitted through electronic alerts to their care team [14]. Additional information on pain and expected length of stay, as well as evidence-based strategies to self-manage identified symptoms, side effects, and aspects of recovery can be provided to patients using multiple formats. For example, education about the benefits of early mobilization and less reliance on strong analgesics may be particularly important in facilitating early recovery [33].

### Step 5: Promote Effective Discharge Planning

Discharge planning that includes appropriate and useful information for patients and their caregivers reduces length of hospital stay and unplanned hospital readmissions, improves quality of inpatient and home care, and increases patient satisfaction [34]. The eHealth programs enable discharge plans to be readily accessible to patients at their own convenience. Information and links to available services and support resources can be tailored to the patient’s condition, location, and procedure. Information about whom to contact and when to contact particular health care providers in the event of complications can also be incorporated.

### Step 6: Optimizing Rehabilitation and Long-Term Follow-Up

The need to undergo additional surgery to manage complications can be minimized through continuity and timeliness of follow-up care. Patients self-reporting symptoms from home through eHealth programs can result in earlier symptom detection, improve communication, and provide an efficient means to capture data evaluating the effects of procedures on health-related quality of life. Interactive health communication apps combine health information with social support, decision support, or behavior change support and can improve knowledge, social support, and behavioral and clinical outcomes [35]. Programs can be designed to enable goal setting, monitoring of progress, and tailoring of recommendations regarding activities and resources that may be helpful to achieve goals.

These programs can also reduce the burden associated with travel and accommodation for follow-up care. For example, the current practice of routine, face-to-face follow-up of patients who received asymptomatic total joint replacement may be excessively costly and unnecessary. In this situation, tele-rehabilitation via Web-based communication following the surgery may be an alternative option [36], especially for patients who are located remotely. It enables a surgeon to conduct a follow-up consultation without being physically present using a mobile remote videoconferencing equipment.

## Challenges of eHealth and Future Directions

### Overview

While promising, a number of potential disadvantages to eHealth programs have been raised in relation to inequity in access to the Internet, poor health literacy, and concerns over privacy and costs. The notion of a “digital divide” in relation to access has been highlighted for particular subgroups, such as those residing in rural areas [37]. Similarly, older people report lower rates of Internet use [38]. As the demand for orthopedic, cardiovascular, and cancer surgery increases as a consequence of an aging population, these access issues must be considered when proposing eHealth programs [39-41]. Others express concern that some groups might have less capacity for eHealth programs. Poor health literacy and cognitive deficits in end users may be particularly challenging. However, integrating features, such as presenting information in a range of accessible formats such as video and audio clips, may help overcome these issues. Familiarity with e-technology is increasing, with growing mobile phone and tablet ownership, which suggests its acceptability in day-to-day life. Research also shows that these are acceptable to people from a variety of health care settings, including surgical patients.

### Internet-Based Interventions Are Promising but More Evidence Is Needed

The Internet has been touted as promising for diverse applications in surgical patients’ care, such as real-time monitoring lifestyle behaviors among candidates for bariatric surgery [42], and educating breast augmentation patients regarding treatments, medications, and surgical options [43]. However, there is limited evidence of the impact of such approaches on patient outcomes. This may to some extent reflect reluctance to test online interventions in those cases where the evidence for the intervention delivered by more conventional means (eg, face to face) is mixed or ambiguous. For example, there is mixed evidence that face-to-face and telephone-delivered preoperative interventions for surgical patients can improve a number of outcomes such as knowledge, pain, recovery time, and anxiety [26,30,44-46]. The mixed nature of research findings likely suggests that the specific nature of the intervention (content and dose) and the specific patient population need to be considered when making judgments about intervention effectiveness.

There have also been limited studies that evaluated the impact of online preparatory interventions on patient outcomes or processes of care. One randomized controlled trial showed that orthopedic patients who received Internet-based education on anesthesia options before surgery had greater knowledge of anesthesia and were more likely to choose neuraxial rather than general anesthesia compared with the control group [47]. Similarly, although there is emerging evidence that interactive eHealth interventions have positive effects on knowledge, social support, and potentially on behavioral and clinical outcomes for people with chronic diseases [48], few studies have examined the impact of Internet-delivered interventions for improving self-management and recovery in the perioperative period.

The current generation of mobile phones provides access to Internet [49] with wireless capabilities enabling users to have continuous access from any location [50]. Such continuous connectivity holds immense potential for use in health care [49] and the use of mobile technology in patient care is particularly appealing [51] because of its portability, continuous uninterrupted data stream, and capability to support multimedia software apps [49]. The mobile app industry is also rapidly evolving [51] with a huge potential for interventions to benefit health and health service delivery processes. For example, a previous study reported that for low-risk postoperative ambulatory patients, use of a mobile app for follow-up care was suitable [52]. Although a range of surgical mobile phone apps exist that could benefit both surgeons and patients [53], systematic reviews on the impact of such technologies on health outcomes remain scarce [50]. Interdisciplinary collaboration is thus essential for future advances in this field [51].

### Gap Between the Interest in eHealth Educational Tools and Real-World Usage

The eHealth programs have the potential to enable a dramatic transformation in the delivery of surgical care, making it safer, more effective, and more efficient. However, in order for eHealth interventions to achieve these goals, they must be accessible to and used as intended by consumers. Therefore, it is imperative that strategies to maximize consumer engagement and uptake of eHealth programs be considered in any intervention trials. When designing such eHealth programs for surgical patients, key learnings from other areas in which eHealth has been successfully applied may be useful to consider. For example, a meta-analysis showed that online health behavior interventions that are brief, goal oriented, and include tools or strategies to show users the consequences of their actions, assist them in meeting goals, and apply normative social pressures are more likely to be adhered to than those without these features [54]. Another review found that eHealth interventions that include greater interaction with a health care provider, greater dialogue support (eg, praise, peer examples), and more frequent updates were likely to be adhered to by participants [55]. While the impact of eHealth programs is usually measured based on a specific population (eg, people undergoing knee replacement surgery), it is important that the influence of other factors, such as geographical location, are also considered, as these may confound findings. Although it is unclear whether such factors will influence surgical patients’ adherence and engagement to eHealth interventions in the absence of surgery-specific studies, these provide a useful starting point.

### Augmenting Surgical Care Across the Entire Surgical Care Pathway

Most research on eHealth has focused on improving care during one specific phase of the surgical care pathway, such as preoperative preparation or discharge planning. Segmenting surgical care in this manner does not mirror the patient’s experience. Poor patient outcomes may be a consequence of the type of care received during a particular phase on the continuum (eg, suboptimal consent process) or the transition between different phases (eg, transfer between hospital and home/community services). Targeting improvement strategies to a single phase does not acknowledge the interdependence between each phase. Thus, eHealth programs that promote a holistic model of care across the entire surgical care pathway should be considered.

### Promoting a Dyadic Approach to Surgical Care

Despite the increased reliance on family and friends to provide informal care for surgical patients, carers often feel unprepared for the patient’s transition from hospital to home. Inadequate preparation results in poorer physical health and high levels of perceived strain and disruptions to family and social life. The eHealth programs can deliver information about strategies that the carer can implement to assist the patient, including how to assist with daily living activities, monitor emotional well-being, and when to contact services for help. Programs can also provide information and activities that the carer can utilize to help manage their own well-being.

### Conclusions

The eHealth platforms have the potential to address gaps in the gathering and transfer of information across the 6 phases of the surgical journey. Rather than approaching each of these phases as separate entities, interventions should strive to address each of the phases to promote continuity and holistic care. Rigorous intervention studies are needed to determine the impact of these programs on patient outcomes and processes of care. Studies examining the role of eHealth programs in supporting carers, and strategies to maximize engagement from end users are also needed.
